# Epidemiology and Ecology of Opportunistic Premise Plumbing Pathogens: *Legionella pneumophila*, *Mycobacterium avium*, and *Pseudomonas aeruginosa*

**DOI:** 10.1289/ehp.1408692

**Published:** 2015-03-20

**Authors:** Joseph O. Falkinham, Elizabeth D. Hilborn, Matthew J. Arduino, Amy Pruden, Marc A. Edwards

**Affiliations:** 1 Department of Biological Sciences, Virginia Tech, Blacksburg, Virginia, USA; 2 National Health and Environmental Effects Research Laboratory, Office of Research and Development, U.S. Environmental Protection Agency, Research Triangle Park, North Carolina, USA; 3 Centers for Disease Control and Prevention, Atlanta, Georgia, USA; 4 Department of Civil and Environmental Engineering, Virginia Tech, Blacksburg, Virginia, USA

## Abstract

**Background:**

*Legionella pneumophila, Mycobacterium avium*, and *Pseudomonas aeruginosa* are opportunistic premise plumbing pathogens (OPPPs) that persist and grow in household plumbing, habitats they share with humans. Infections caused by these OPPPs involve individuals with preexisting risk factors and frequently require hospitalization.

**Objectives:**

The objectives of this report are to alert professionals of the impact of OPPPs, the fact that 30% of the population may be exposed to OPPPs, and the need to develop means to reduce OPPP exposure. We herein present a review of the epidemiology and ecology of these three bacterial OPPPs, specifically to identify common and unique features.

**Methods:**

A Water Research Foundation–sponsored workshop gathered experts from across the United States to review the characteristics of OPPPs, identify problems, and develop a list of research priorities to address critical knowledge gaps with respect to increasing OPPP-associated disease.

**Discussion:**

OPPPs share the common characteristics of disinfectant resistance and growth in biofilms in water distribution systems or premise plumbing. Thus, they share a number of habitats with humans (e.g., showers) that can lead to exposure and infection. The frequency of OPPP-infected individuals is rising and will likely continue to rise as the number of at-risk individuals is increasing. Improved reporting of OPPP disease and increased understanding of the genetic, physiologic, and structural characteristics governing the persistence and growth of OPPPs in drinking water distribution systems and premise plumbing is needed.

**Conclusions:**

Because broadly effective community-level engineering interventions for the control of OPPPs have yet to be identified, and because the number of at-risk individuals will continue to rise, it is likely that OPPP-related infections will continue to increase. However, it is possible that individuals can take measures (e.g., raise hot water heater temperatures and filter water) to reduce home exposures.

**Citation:**

Falkinham JO III, Hilborn ED, Arduino MJ, Pruden A, Edwards MA. 2015. Epidemiology and ecology of opportunistic premise plumbing pathogens: *Legionella pneumophila, Mycobacterium avium,* and *Pseudomonas aeruginosa*. Environ Health Perspect 123:749–758; http://dx.doi.org/10.1289/ehp.1408692

## Introduction

Because community water systems supply water to > 95% of the approximately 300 million people in the United States, there is a need for a firm understanding of the precise contribution of these water systems to the spread of waterborne disease (Craun et al. 2010; Kozicki et al. 2012). Community water systems deliver water to premise plumbing, which is the portion of the water distribution system beyond the property line and includes households, office buildings, and hospitals. Therefore, premise plumbing serves as the interface of exposure of people to the microbes inhabiting their water supply.

Several unique features of premise plumbing can increase risk of microbial infection. High surface-to-volume ratio, intermittent stagnation, low disinfectant residual, and warming cycles can stimulate growth of waterborne pathogens. High surface-to-volume ratios of premise plumbing means that there is a large area conducive for biofilm formation. Biofilms can be attractive habitats for pathogens and offer protection from disinfectants. Opportunistic pathogens have been found to grow in shower heads, faucets, along pipe walls, and in water heaters. The long residence time of water in premise plumbing enhances biofilm formation, including growth of resident pathogens. Although greater water ages are thought to enhance attenuation of traditional enteric pathogens, opportunistic pathogens can adapt and grow at low oxygen levels characteristic of stagnation in premise plumbing.

Herein, we review the epidemiology and ecology of opportunistic premise plumbing pathogens (OPPPs), focusing on three of the most commonly tracked bacterial agents: *Legionella pneumophila, Mycobacterium avium*, and *Pseudomonas aeruginosa*. Infections by all three have been linked to human exposure via premise plumbing (Table 1). It has been estimated that the costs of the estimated 29,636 cases of OPPP disease per year is approximately $850 million (Collier et al. 2012).

**Table 1:** Estimated OPPP disease occurrence.

Disease (ICD-10 code)	Measurement	Source
Disease prevalence/100,000
Legionnaires’ (A48.1)	0.39 (2000) to 1.36 (2011)	CDC 2011b, 2013a
NTM*, *USA (031.0)		
Men	3.5 (1994–1996) to 4.9 (2004–2006)	Prevots et al. 2010
Women	4.5 (1994–1996) to 7.5 (2004–2006)	Prevots et al. 2010
NTM, Ontario (031.0)	29.3 (1998–2002) to 41.3 (2006–2010)	Marras et al. 2013
Isolation prevalence/year
*P. aeruginosa* (J15.1)	2,025 (1986) to 5,205 (2000)	Gaynes et al. 2005
*Acanthamoeba*	> 3,000 global cases (2004)	Schuster and Visvesvara 2004
*N. fowleri*	111 cases (1962–2008)	Yoder et al. 2008
Abbreviations: ICD-10, *International Classification of Diseases, 10th Revision*; NTM, nontuberculous mycobacteria.		

Until recently [e.g., 1976 (*Legionella*), 1980 (*Mycobacterium*), 1997 (*Pseudomonas aeruginosa*)], there was no consideration of pathogenic microorganisms that were natural inhabitants of water and drinking water systems. In the past, the term “waterborne pathogens” referred to those agents in human or animal waste that entered water as contaminants with ingestion of drinking water, the principle route of exposure and infection. Classically, these included polio virus, *Shigella, Salmonella*, and other enteric bacteria, all examples of pathogens with a fecal–oral route of infection. Those pathogens are not normal inhabitants of water but contaminants, and generally are not capable of reproduction in the water supply. Operationally, one can identify the point source for such “waterborne pathogens” by moving upstream as their numbers increase as they get closer to the point source. In contrast, the numbers of OPPPs increase as the distance from the treatment plant increases because they can multiply in pipes and plumbing systems (Falkinham et al. 2001; Hardalo and Edberg 1997; Hsu et al. 1984; Lin et al. 1998; Squier et al. 2000; States et al. 1987). Further, OPPP numbers do not correlate with fecal coliform numbers (Falkinham et al. 2001).

Increasingly, the role of biofilms within water distribution systems and premise plumbing is recognized as important for the establishment and maintenance of the chronic colonization associated with *L. pneumophila, M. avium*, and *P. aeruginosa*. Biofilms and amoebic host organisms can protect these pathogens from effective treatment with disinfectants, such as chlorine (Donlan and Costerton 2002; Høiby et al. 2010; Murga et al. 2001; Simões et al. 2010; Steed and Falkinham 2006; Suman et al. 2008).

On 25–27 March 2012, a Water Research Foundation–sponsored expert workshop, “Research Needs for Opportunistic Pathogens in Premise Plumbing,” was held at the Virginia Tech Northern Virginia Center, Falls Church, Virginia, to document the widespread prevalence and impact of OPPPs on humans and to review their epidemiology and ecology and identify common features contributing to the infective process. The 2-day workshop assembled > 50 experts in drinking water and waterborne pathogens to *a*) review the state of knowledge of OPPPs, *b*) identify gaps in knowledge of OPPPs, and *c*) identify and prioritize research objectives (Pruden et al. 2013). This literature review serves to summarize the state of the knowledge with respect to epidemiology and ecology of key bacterial OPPPs, which may better inform the development of approaches to reduce human exposure and infection. Key knowledge gaps identified at the expert workshop that should be prioritized for future research are also described.

## *Legionella* Epidemiology

There are three major presentations of *Legionella* spp. infection: *a*) Pontiac fever and *b*) either community-acquired or *c*) outbreak-associated Legionnaires’ disease. Pontiac fever is an influenza-like, mild illness that is spontaneously resolved without therapy and is caused by a variety of *Legionella* spp. from either water (Castor et al. 2005) or soil (Cramp et al. 2010). The focus here will be on *L. pneumophila*, the causative agent of serious, life-threatening pneumonia (“Legionnaires’ disease”), often requiring hospitalization [Centers for Disease Control and Prevention (CDC) 2011b; Yoder et al. 2008]. In the United States, the most recent estimated number of hospitalized cases, based on a population-based study in 2000–2009, is 8,000–18,000 (CDC 2011b). Moreover, the number of reported legionellosis cases increased 3.5-fold between 2000 and 2011 (Table 1); (CDC 2011b, 2013a; Yoder et al. 2008). Legionellosis is an acute illness and, in its most severe form, is generally responsive to timely and appropriate antimicrobial therapy (Bruin et al. 2012; Niederman et al. 2001).

In addition to air-conditioning systems and cooling towers as sources (Nguyen et al. 2006; Sabria et al. 2006), it is now understood that drinking water is an important source of *L. pneumophila* (Benin et al. 2002; Boccia 2006; CDC 2013b; Neil and Berkelman 2008; Phares et al. 2007; Yoder et al. 2008). *L. pneumophila* recently became the single most common cause of reported disease outbreaks involving drinking water (CDC 2013b; Craun et al. 2010; Yoder et al. 2008), in part because of improved detection and diagnosis and also likely because it is the first OPPP-associated disease that must be reported to the National Notifiable Diseases Surveillance System (http://wwwn.cdc.gov/nndss/).

*Community-acquired* L. pneumophila *pneumonia.* The prevalence of community-acquired and healthcare-associated legionellosis are both increasing. One-quarter (25%) of *Legionella* spp. infections are healthcare associated (Neil and Berkelman 2008). Because *L. pneumophila* is present in drinking water distribution systems and household water (Arnow et al. 1985; Bollin et al. 1985; Borella et al. 2005; Donohue et al. 2014), persons at risk for Legionnaires’ disease should take precautions. Risk factors for Legionnaires’ disease include reduced immune competence, smoking, alcoholism, and older age (CDC 2011b). Case reports are highest in summer and in the mid-Atlantic region of the United States (CDC 2011b).

*Outbreak-associated* L. pneumophila *pneumonia*. Legionnaires’ disease was first described as an outbreak of pneumonia amongst attendees of an American Legion convention in Philadelphia, Pennsylvania, in 1976 (Fraser et al. 1977). In the absence of evidence of person-to-person transmission, it was hypothesized that the infective agent originated from the environment (Fraser et al. 1977). Since that first report, outbreaks of *L. pneumophila* disease have been linked to water sources in hospitals, hotels, cruise ships, industrial facilities, and multiple and single family residences (Arnow et al. 1985; Borella et al. 2005; Hung et al. 1993; Kusnetsov et al. 2003; O’Loughlin et al. 2007; Polverino et al. 2010). The British Communicable Disease Surveillance Centre reported that 19 of 20 hospital outbreaks of Legionnaires’ disease in the United Kingdom from 1980 to 1992 were primarily attributed to hospital water systems (Joseph et al. 1994). *Legionella* spp. in hospital drinking water samples have been linked to patient isolates by DNA-fingerprinting methods (Den Boer et al. 2008; Kozak-Muiznieks et al. 2014; Ragull et al. 2007; Sabrià et al. 2001; Stout et al. 1985) Consequently, Legionnaires’ disease should be considered for all pneumonia cases with prior hospital exposure, particularly the elderly, smokers, immunosuppressed, and those with chronic lung disease.

## *Legionella* Ecology

The natural habitat for *Legionella* appears to be aquatic bodies including rivers, streams, and thermally polluted waters (Brooks et al. 2004; Colbourne and Dennis 1989; Hsu et al. 1984; Lin et al. 1998; Stout et al. 1985). *Legionella* bacteria have been detected in all segments of water distribution—from the source water (rivers and groundwater) to the tap. Natural aquatic bodies contain only small numbers of *Legionella*. The presence of *Legionella* in a water distribution system is not necessarily an indication that the system is poorly maintained because this bacterium may be a normal constituent of the microbial population of water distribution systems. It has been estimated that *Legionella* are found in approximately 50% of large building water systems and 10–30% of home water systems in the United States (Kool et al. 1999; Stout and Yu 2011), and detection methods are becoming increasingly sensitive. Depending on the study and methods, a range of 12–70% of hospital water systems are estimated to be colonized with *Legionella* (Stout and Yu 1997). A recent publication demonstrated the presence of “nonculturable” cells of *L. pneumophila* as well as methods for their resuscitation, to ensure that colony counts are not underestimated (Ducret et al. 2014). In the first national study to use more sensitive molecular techniques, *Legionella* genetic material was detected in 50% of cold water samples (Donohue et al. 2014).

Cooling towers and, to a lesser degree, evaporative condensers were implicated in the earlier outbreaks prior to recognition of potable water as a reservoir (Bentham 2000; Nguyen et al. 2006). The emphasis of cooling towers in the dissemination of *Legionella* has been challenged (Stout and Muder 2004). Reports of cooling towers as reservoirs for legionellosis have dwindled in comparison with those linked to building water distribution systems.

*Legionella* are not completely eliminated from drinking water by standard water treatment practices. For example, *Legionella* are comparatively more resistant to chlorine than *Escherichia coli* (Garcia and Pelaz 2008; Hosein et al. 2005; Kim et al. 2002; Zhang et al. 2007). *Legionella* are also known to be sheltered within encysted amoebae; indeed, after phagocytosis by amoebae, whose cells are relatively chlorine-resistant, *Legionella* can survive up to 50 ppm chlorine (Kilvington and Price 1990). *Legionella* growth and proliferation occur in engineered habitats, especially water distribution systems, which provide favorable water temperatures (25–42°C), surfaces for biofilm formation, and nutrients (Arnow et al. 1985; Donlan and Costerton 2002; Lin et al. 1998; Murga et al. 2001). One important factor appears to be water temperature. Buildings with recirculating hot water distribution systems colonized with *L. pneumophila* were significantly more likely to have lower hot water heater temperatures (< 60°C) than systems that were not colonized (Arnow et al. 1985; Darelid et al. 2002). The microorganism is readily found in biofilm and detritus at the bottom of hot water tanks. Bacteria, protozoa, and amoebae also colonize water pipe surfaces, some of which have been shown to promote *Legionella* replication (Buse et al. 2014b; Kilvington and Price 1990). *Legionella* and other microorganisms attach to surfaces and form biofilms on pipes throughout the water distribution system. Cold-water sources, such as ice from ice machines and water from fountains with stable biofilm-colonized surfaces, have also been implicated as a source of infection (Hoebe et al. 1998; O’Loughlin et al. 2007; Stout et al. 1985).

## Sources of *Legionella* Exposure and Transmission

Multiple modes have been identified for transmission of *Legionella* to humans; there is evidence for aerosolization, aspiration, or even instillation into the lung during respiratory tract manipulation. Because one of the first environmental isolations of *L. pneumophila* was from a showerhead (Stout and Yu 2003), it has been widely thought that aerosols from showers may be an important means for dissemination of this microorganism. However, as *Legionella* are prevalent in home water systems, any shower or faucet can be a source of infection (Stout and Muder 2004), and detectable airborne *Legionella* aerosols have been detected in proximity to faucets (Bollin et al. 1985).

Aspiration of contaminated water or oropharyngeal secretions appears to be the major mode of transmission in the hospital setting (Blatt et al. 1993; Yu 1993). Colonization of oropharyngeal flora by *L. pneumophila* is a theoretical possibility, and the evidence for aspiration has accumulated. Nasogastric tube placement has been shown to be a significant risk factor for healthcare-associated legionellosis in intubated patients; microaspiration of contaminated water was the presumed mode of entry (Blatt et al. 1993). It is possible that ingestion of water also can play a role. During the original 1976 outbreak, consumption of water and possible aspiration at the implicated hotel was associated with acquisition of disease (Fraser et al. 1977)—an association that has been generally overlooked.

Healthcare personnel frequently use tap water to rinse respiratory apparatus and tubing used for ventilators. If the tap water contains *L. pneumophila*, the bacteria could possibly be instilled directly into the lung of a patient (Tablan et al. 2004). In numerous studies, the risk of Legionnaires’ disease was significantly greater for patients who underwent endotracheal tube placement more often or had a significantly longer duration of intubation than for patients who had other causes of pneumonia. Use of sterile water for all nasogastric suspensions, for humidifiers in breathing circuits of mechanical ventilators, and for flushing tubes has been recommended to prevent *Legionella* infection (Tablan et al. 2004).

## *Mycobacterium avium* Epidemiology

*M. avium* infections are known to originate from environmental sources (Falkinham 1996). It is one among 175 species of the genus *Mycobacterium* that do not belong to the *Mycobacterium tuberculosis* complex (Tortoli 2003) and thus are called nontuberculous mycobacteria (NTM). Our focus is on *M. avium* as the causal agent of the majority of NTM infections in the United States (Falkinham 1996), and it is the most prevalent *Mycobacterium* in drinking water (Falkinham 2011; Falkinham et al. 2001).

There are three major presentations of *M. avium* infection: *a*) bacteremia in HIV-infected individuals, *b*) cervical lymphadenitis in young children, and *c*) community-acquired *M. avium* infection in adults. There have been few reports of *M. avium* disease outbreaks, and these tend to be associated with contamination of solutions and instruments in hospitals (Wallace et al. 1998). Reports have also linked isolation of *M. avium* from bronchoscopes, ice, whirlpool tubs, pools, footbaths, and prepared cleaning and irrigation solutions (Gubler et al. 1992; Kahana et al. 1997; Winthrop et al. 2002). *M. avium* bacteremia among HIV-infected and immunosuppressed persons emerged during the 1980s (Horsburgh and Selik 1989). At one time, approximately 50% of late-stage AIDS patients had *M. avium* bacteremia (Horsburgh and Selik 1989), but with the implementation of highly active antiretroviral therapy, the number of HIV-infected patients with *M. avium* infections has fallen dramatically. Although there are no national statistics documenting numbers of *M. avium*–associated cervical lymphadenitis in children, there has been no published evidence of this manifestation disappearing or increasing (Wolinsky 1995). In a comprehensive, prospective study of cervical lymphadenitis published in 1995 by a physician evaluating incident cases in the United States over the period of 1958–1990, only 105 cases were found (Wolinsky 1995). The age of the infected children (median age 3 years) suggests that exposure to water or soil containing *M. avium*, coupled with gum trauma due to erupting teeth, led to *M. avium* infection of the lymph nodes of the head and neck.

*Community-acquired* M. avium *infection*. Currently, the majority of *M. avium* cases are community acquired. As disease caused by *M. avium* is not nationally notifiable in the United States (CDC 2011a), population-based studies are uncommon, and the public health burden of *M. avium* disease is difficult to measure. Two recent population-based studies in the United States have described rates of *M. avium* disease as increasing among older persons and women (Table 1) (Prevots et al. 2010; Winthrop et al. 2010). Estimated prevalence of *M. avium* lung disease varies by study, location, age, and susceptible population; prevalence increases with age and is highest among patients with AIDS, at 647 cases/100,000 persons (Marras and Daley 2002; Prevots et al. 2010).

Among studies that evaluated numbers of *M. avium* isolates recovered from clinical specimens, rates of positive cultures appear to be increasing. These include reports from the United States (du Moulin et al. 1985; Prevots et al. 2010), Japan (Tsukamura et al. 1988), Canada (Al Houqani et al. 2011; Marras et al. 2013), Taiwan (Chen et al. 2011), South Korea (Ryoo et al. 2008), and China (Wang et al. 2010). The increase in *M. avium* isolation and disease frequency has also coincided with a shift in the observed epidemiology, from disease occurring principally in older men with reduced lung function due to smoking or occupational dust exposure, to tall, slender, and older women (Prince et al. 1989). Among studies where age is reported, older age was associated with a higher prevalence of disease. However, factors (e.g., improved detection) other than age alone may be associated with increased rates reported per year in many studies, even in countries with aging populations (Al-Houqani et al. 2012).

Both pulmonary and extrapulmonary *M. avium* infections have been described (Bodle et al. 2008; Marras and Daley 2002; Winthrop et al. 2002). Extrapulmonary sites of *M. avium* infection include skin and soft tissue, catheter-related bacteremia, exit-site infection, surgical site infections, and infections such as peritonitis, lymphadenitis, keratitis, and osteomyelitis (Piersimoni and Scarparo 2009). *M. avium* disease typically involves subacute to chronic infections that are difficult to treat and are frequently resistant to antimicrobials (Brown-Elliott et al. 2012; Griffith et al. 2007; Philley and Griffith 2013). Susceptibility to *M. avium* disease is poorly characterized; however, multiple host risk factors have been associated with *M. avium* infection. Competent cell-mediated (innate) immunity is an essential first line of defense against mycobacterial infections; defects in immune response pathways or in T-cell function may be a risk factor for infection (Collins 1989; Holland 2007). Chronic lung diseases, such as chronic obstructive pulmonary disease, bronchiectasis (Fowler et al. 2006; Maugein et al. 2005), silicosis (Armen and Morrow 1956; Bailey et al. 1974), cystic fibrosis (Olivier et al. 2003), alveolar proteinosis (Witty et al. 1994), pneumoconiosis (Fujita et al. 2004), and previous tuberculosis (Sonnenberg et al. 2000), have all been identified as risk factors for NTM-associated pulmonary disease. Other risk factors for NTM pulmonary disease include connective tissue disorders and abnormal cystic fibrosis genotypes (Iseman et al. 1991; Kim et al. 2008).

Multiple reports have linked *M. avium* disease with exposure to drinking water, using molecular typing methods. Closely related *M. avium* isolates have been recovered from hospitalized patients and water in the hospitals (Tobin-D’Angelo et al. 2004; von Reyn et al. 1994), from residents and their household water (Falkinham 2011; Falkinham et al. 2008), and from a resident and his/her municipal water (Hilborn et al. 2008). Strains of *M. avium* isolated from humans have been shown to be competent biofilm formers (Carter et al. 2003; Mullis and Falkinham 2013; Williams et al. 2009). An *in vitro* study suggested that a *M. avium* strain’s ability to form biofilm is associated with pathogenicity and invasion of bronchial epithelial cells (Yamazaki et al. 2006).

## *Mycobacterium avium* Ecology

Many factors appear to interact in complex and poorly characterized ways to support or inhibit *M. avium* growth in water pipes. Factors include temperature, water flow, nutrients, pipe material and condition, residual disinfectant, free-living phagocytic amoebae, mycobacteriophages, and other bacteria. Reports implicate some of these risk factors, but none alone predict *M. avium* concentration at the point of use. Concentrations of *M. avium* in water were significantly correlated with organic carbon concentrations (Falkinham et al. 2001), hot water plumbing lines (du Moulin et al. 1988; Falkinham 2011), and plastic pipe material (Schulze-Röbbecke et al. 1992), although Norton et al. (2004) reported significant *M. avium* concentrations in water independent of pipe material.

*M. avium* has been isolated from multiple environmental sources, including from water and biofilm (Schulze-Röbbecke et al. 1992; Tsintzou et al. 2000). Reports of *M. avium* in water distribution and treatment plants suggest that biofilms that form in the distribution system act as an important niche for the survival of *M. avium* (Falkinham et al. 2001; Feazel et al. 2009; Hilborn et al. 2006). Further, *M. avium*, like *L. pneumophila* and *P. aeruginosa*, is an amoeba-resisting microorganism, able to grow and survive in amoebae (Cirillo et al. 1997; Thomas and Ashbolt 2011). *M. avium* is approximately 500 times more resistant to chlorine than *Escherichia coli* (Taylor et al. 2000) and 40 times more tolerant to chlorine than *P. aeruginosa* (Grobe et al. 2001). Further, *M. avium* survives and multiplies in distribution systems despite ambient chlorine residual concentrations (Falkinham et al. 2001). *M. avium* grown in water is more chlorine resistant than the same strains grown in culture medium, and most strains are more resistant to chloramine than to free chlorine (Taylor et al. 2000).

Drinking water is a known environmental source of NTM and has been extensively studied in an attempt to characterize the risk of human exposure to NTM. Quantitative interpretation of the results of these studies is problematic because isolation methods vary and because decontamination steps to prevent the growth of more rapidly growing microorganisms are known to reduce concentrations of *M. avium* in water samples (Thomson et al. 2008). Unfortunately, there are no selective or differential media for the cultivation of NTM from samples containing other microorganisms. Therefore, observed and reported occurrence in water samples should be interpreted as conservative estimates of true occurrence and abundance. *M. avium* isolation from drinking water has been documented at points of use within both public and private buildings (Falkinham et al. 2008; Hilborn et al. 2006; Perkins et al. 2009).

## Sources of *M. avium* Exposure and Transmission

Water is a well-documented source of *M. avium* exposure. *M. avium* isolation from hospital water supplies is of particular concern due to the potential for exposure of immunosuppressed patients (Baird et al. 2011; du Moulin et al. 1988). The persistence of a single clone of *M. avium* (up to 18 months) in hospital (von Reyn et al. 1994) and distributed municipal drinking water (Hilborn et al. 2006) as a potential chronic source of human exposure is a major challenge. It is important to point out that identification of identical *M. avium* clones in patients and their household plumbing does not necessarily indicate that the tap water is the original source, especially if the patients collected the samples. It could be that patients continually reinfect their own taps.

Filters have been recommended as a means to reduce *M. avium* exposure from water. However, some types of filters, that is, granular activated charcoal (GAC) filters, have been shown to be colonized by *M. avium* and support their growth (Holinger et al. 2014; Rodgers et al. 1999; Williams et al. 2011). Consequently, the GAC filter becomes a source of *M. avium* and likely the other OPPPs.

## Epidemiology of *Pseudomonas aeruginosa*

There are four major presentations of *P. aeruginosa* infection (Table 1): *a*) bacteremia in immunocompromised individuals, *b*) pneumonia in cystic fibrosis (CF) patients, *c*) community-acquired ear and pneumonia infections, and *d*) hospital-acquired outbreaks, principally associated with contaminated solutions or medical devices used in general patients or those in intensive care units (ICUs) (Fujitani et al. 2011). In all four presentations, water containing *P. aeruginosa* is the source of infection.

*Community-acquired pneumonia*. Very few data are available about the occurrence of *P. aeruginosa* disease outside of the healthcare setting. Much of what is known about infections in the community setting is from reports in the published literature (e.g., case reports, outbreak investigations). Infections produced by *P. aeruginosa* are not nationally notifiable, so the burden of disease is difficult to assess. The primary infections (ear and skin) acquired in the community involve the use of swimming pools, hot tubs, and whirlpools where there has been a failure to maintain the equipment or to maintain sufficient residual disinfectant (Hlavsa et al. 2014). *P. aeruginosa* is rarely carried by healthy individuals (2–10% of individuals, likely in the ear) but can be recovered from 50–60% of hospitalized patients (Cholley et al. 2008). *P. aeruginosa* is a major cause of otitis externa (“swimmer’s ear”), with a magnitude of 2.4 million cases per year and an estimated outpatient cost of approximately $500 million (CDC 2011a).

*Hospital-acquired infections*. In a meta-analysis of 43 water-associated outbreaks in hospitals covering 35 years (1966–2001), it was estimated that there were approximately 1,400 nosocomial pneumonia deaths per year in the United States caused by *P. aeruginosa* (Anaissie et al. 1902). Among gram-negative healthcare-associated pathogens, *P. aeruginosa* is the second most frequent pathogen causing ventilator-associated pneumonia, and the third or fourth most frequent pathogen causing septicemia, urinary tract infections, and surgical wound infections (Trautmann et al. 2009). A 10-year molecular epidemiological study attempted to determine the respective roles of exogenous and endogenous flora and time on infection and the effect of *P. aeruginosa* infection in ICU patients (Cuttelod et al. 2011). Isolates fell into three types: *1*) identical patient and faucet isolates; *2*) identical patient isolates, but none in faucets; and *3*) unrelated patient and faucet isolates. Higher levels of faucet contamination with *P. aeruginosa* were correlated with higher numbers of cases in type 1; namely 34/1,000 patient admissions (Cuttelod et al. 2011). The number of type 3 or “endogenous” cases was considerably lower and stable over time (Cuttelod et al. 2011). Two studies of *P. aeruginosa* transmission among ICU patients, using either pulsed-field gel electrophoresis (PFGE) or arbitrary-primed polymerase chain reaction (AP-PCR), established that patient isolates contaminated faucets (Reuter et al. 2002; Rogues et al. 2007). This information should be considered when hypothesizing transmission pathways involving other OPPPs; for example, are *M. avium*–infected patients the source of identical clones of *M. avium* in household plumbing?

P. aeruginosa *in cystic fibrosis*. Cystic fibrosis (CF) patients become colonized early in life with *P. aeruginosa*, and the prevalence of colonization increases with age (Rajan and Saiman 2002). There are 30,000 cystic fibrosis patients in the United States, and 1,000 new patients are diagnosed annually (Aaron et al. 2010). *P. aeruginosa* is a major pathogen of CF patients; 60–80% of CF patients are infected (Aaron et al. 2010). Although a proportion of CF patients are infected with *P. aeruginosa* from other patients (cohabitating hospital wards), the major source of infection is water (Geddes 2008; Hayes et al. 2010).

## *Pseudomonas aeruginosa* Ecology

*P. aeruginosa* is a ubiquitous gram-negative bacillus commonly associated with soil and water. It has minimal nutritional requirements, which enables it to survive in many different environments; it is especially known for possessing a characteristically large genome with vast metabolic capabilities (Klockgether et al. 2011), and it is a model biofilm-forming organism (Masák et al. 2014). In addition to the ability of pseudomonads to grow on a wide variety of organic compounds, *P. aeruginosa* is resistant to chlorine and other disinfectants used in water treatment (Grobe et al. 2001; Seyfried and Fraser 1980). *P. aeruginosa* has been isolated from water (Favero et al. 1971; Mena and Gerba 2009), antimicrobial soaps (Bertrand et al. 2000; Lanini et al. 2011), as well as disinfectant solutions and chlorine-based sanitizing solutions (Russell 1999). *P. aeruginosa* is widely detected in a variety of aquatic environments, including tap water; it is notoriously subject to multiple antibiotic resistance, and therefore *P. aeruginosa* infections can be very difficult to treat (Vaz-Moreira et al. 2012).

## Sources of *P. aeruginosa* Exposure and Transmission

The role of tap water as a source of *P. aeruginosa* disease has been established in a number of published studies (Crivaro et al. 2009; Trautmann et al. 2001, 2005, 2009). The modes of transmission have included direct contact with water and aerosols, aspiration, indirect transfer from moist environmental surfaces, and via healthcare worker hands (Döring et al. 1993; Hollyoak et al. 1995). Several studies used molecular markers to demonstrate relatedness of tap water and patient isolates. Tap water samples from sinks within ICUs have been found to contain *P. aeruginosa* strains that were identical by molecular typing to those obtained from infected and colonized patients (Crivaro et al. 2009). Tap water faucets were colonized with the same *P. aeruginosa* strain for > 2 years, even though *P. aeruginosa* was not recovered from the mains supplying the sinks (Reuter et al. 2002). Tap water and outlets appear to be the reservoir for *P. aeruginosa* within healthcare facilities (Trautmann et al. 2001, 2005, 2009). However, in a surveillance study of hospitalized patients, most were colonized before admission (Cholley et al. 2008). Based on the identity of isolates from both water and patients, *P. aeruginosa* in tap water was found to be the source of infection in 1 of 14 patients (Cholley et al. 2008).

## Future of OPPPs

Information available for disease incidence for each bacterial OPPP that we focus on in this review are listed in Table 1, with two amoebal OPPPs also indicated for comparison as emerging and serious health threats. In particular, *Naegleria fowleri*, a brain-eating amoeba that prefers warm aquatic environments, was recently detected in premise plumbing and linked to high-profile deaths in the southern United States (Bartrand et al. 2014), with investigations ongoing. Overall, it is expected that the number of people susceptible to infection with OPPPs will grow in the United States. Cystic fibrosis patients, transplant recipients, and immunosuppressed patients are living longer lives, resulting in more time to become colonized or infected. For example, the U.S. population is aging, and it is estimated that the proportion of individuals > 60 years of age will increase from 16.1% in 2000 to 24.8% in 2025 (United Nations Population Division 2002). Such an increase in individuals > 60 years of age, coupled with higher rates of infection by OPPPs in that age group, strongly suggests that the prevalence of OPPP-related disease will rise. Our public water systems are deteriorating and in desperate need of maintenance and replacement; thus, there are increased opportunities for intrusion of soil-associated OPPPs and for biofilm formation, which favors their growth. At the same time, exposure occurs via premise plumbing, and knowledge of the interaction between the chemistry and microbiology of municipal water and premise plumbing is needed to inform risk management strategies.

## Common Features of OPPPs

There are several traits shared by the three bacterial OPPPs that select for their presence and persistence in premise plumbing. Those traits include disinfectant resistance, biofilm formation, survival at high temperatures, and growth in free-living phagocytic amoebae. The concentrations of water treatment disinfectants (e.g., chlorine, chloramine) required to kill 99.9% of the bacterial OPPPs are higher than those needed to obtain a 3-log reduction in *Giardia lamblia* cysts (U.S. Environmental Protection Agency 2010), the standard used for water disinfection. The presence of residual disinfectant provides these resistant OPPPs a competitive advantage (Grobe et al. 2001; Seyfried and Fraser 1980; Taylor et al. 2000). One factor likely leading to the occurrence and persistence of the bacterial OPPPs in premise plumbing and distributions systems is their ability to adhere to surfaces and form biofilms. Bacteria in biofilms are also more resistant to disinfectants (Simões et al. 2010). Residence in biofilms also makes bacterial OPPPs more accessible to free-living, phagocytic amoebae [e.g., *Acanthamoeba* and *Vermamoeba* (formerly called *Hartmanella* (Smirnov et al. 2005)], which can actually enhance their proliferation in drinking water (Thomas and Ashbolt 2011). All three bacterial OPPPs belong to the category of amoeba-resisting microorganisms: they are not necessarily killed by amoebae following phagocytosis but can actually survive and grow. Finally, because these OPPS have relative resistance to high temperatures that are encountered in hot water pipes (e.g., 35–45°C), their numbers actually increase in hot water heaters and premise plumbing.

## Remediation and Control of OPPPs

Currently, there are no documented broadly effective community-level engineering control strategies for OPPPs in municipal drinking water or premise plumbing. Identification and use of methods to effectively and economically control OPPPs is in its infancy. Preventative measures could be imposed by water utilities, healthcare operators, and homeowners (Table 2). Although a simple increase in disinfectant concentrations is possible, it would likely be counterproductive in that it would create taste and odor problems, and additional chlorination may result in potentially carcinogenic disinfection by-products. In addition, increasing the disinfection concentrations might be ineffective because the OPPPs are very resistant to disinfectants, particularly those encased within amoebae cysts (Hardalo and Edberg 1997).

**Table 2:** Possible measures to reduce OPPP numbers in premise plumbing.

Measures to be taken	Outcome measure
Water provider action
Reduction of turbidity	Turbidity measurement
Reduction of biologically available carbon	AOC or BDOC measurement
Biofilm-discouraging pipes	Biofilm mass and microbial number reduction
Building manager or homeowner action
Raise hot water heater temperature	Temperature in hot water heater or tap
Employ point-of-use filters at taps and showers	Frequency of POU filters
Increase oxygen levels	Frequency of installed aerators
	Plumbing water oxygen levels
Abbreviations: AOC, assimilable organic carbon; BDOC, biodegradable dissolved organic carbon.	

Participants of the workshop, “Research Needs for Opportunistic Pathogens in Premise Plumbing,” took the view that novel approaches are needed: for example, manipulation of the chemistry or microbiome of drinking water distribution systems and premise plumbing. A recent review identified specific challenges for in-building control of *Legionella*, much of which is driven by limitations of water chemistry, scaling, and corrosion control (Rhoads et al. 2014). Even if possible measures are identified, there may be wide variance in outcomes because of differences in types of disinfectant, organic carbon levels, pipe composition, system design, water chemistry, and even bacterial species and strains. At the level (and responsibility) of the water provider, reduction of turbidity and organic carbon levels and the employment of biofilm-discouraging pipes (e.g., antimicrobial coated or impregnated) might prove useful (Table 2). For the hydrophobic *M. avium* and other mycobacteria, turbidity reductions do reduce numbers (Falkinham et al. 2001), most likely as the cells enter the treatment plant adhered to soil particulates. Reducing organic carbon might also be expected to reduce OPPP concentrations because all three of the species discussed herein are heterotrophs, requiring organic carbon for growth. Although speculative, it might be possible to reduce OPPP biofilm numbers by pretreating distribution and plumbing pipes with agents (e.g., antimicrobials or surfactants) that reduce adherence and biofilm growth.

Individuals or building managers can also employ measures to reduce OPPP numbers in premise plumbing (Rhoads et al. 2014) (Table 2). First, temporary high disinfectant concentrations or high-temperature water can be applied to a building plumbing system to kill viable OPPPs. Although that has been performed successfully, the OPPPs occasionally return, sometimes in higher numbers than were present before the temporary treatment—likely due to necrophilic growth (Temmerman et al. 2006). Point-of-use microbiological filters have been used in some healthcare settings to prevent exposure of patients to OPPPs or to prevent contamination of solutions that may come in contact with patients (e.g., dye, disinfecting solutions). However, those filters can, over time, become sources of OPPPs (Rodgers et al. 1999).

## Research Needs for OPPPs

One outcome of the 2012 expert workshop sponsored by the Water Research Foundation was a listing of research gaps in our understanding and knowledge of the bacterial OPPPs (Pruden et al. 2013). Those research gaps specifically focused on epidemiology and ecology of OPPPs are listed in Appendix 1 and are ordered by priority as judged by the experts participating in the workshop. Of importance above all research needs identified at the workshop was the determination of the prevalence, incidence, and trends of disease caused by OPPPs. Because disease caused by *M. avium* and *P. aeruginosa* is not required to be reported, the full impact of disease caused by these OPPPs can only be estimated—and those are underestimates. In addition, it will be important to determine the relationship between OPPP densities (infectious dose) and disease. Two factors make such risk–analysis calculation difficult: The OPPPs vary widely in virulence (even within single species) and individuals vary widely in susceptibility.

Although the focus of the workshop and this review is on three OPPPs, there are other opportunistic pathogens in premise plumbing. As mentioned above, amoebal OPPPs, such as *N. fowleri* and *Acanthamoeba*, are of growing concern both as pathogens and as hosts for bacterial OPPPs. Other important bacterial OPPPs include *Acinetobacter* spp., *Stentrophomonas* spp., *Brevundimonas* spp., *Sphingomonas* spp., and *Chryseobacterium* spp. (Baron et al. 2014). In addition, the slow-growing, lipid-rich, mycobacterial-like *Segniliparus* spp. isolated from cystic fibrosis patients (Butler et al. 2007) are also waterborne OPPPs.

One novel research concept that was identified by the workshop was the possibility that the microbiome of a water distribution system or premise plumbing could be manipulated to influence the presence of OPPPs. Studies of that type might provide understanding of the distribution system microbiome to aid in predicting when colonization by OPPPs would be favored. This idea was reviewed recently by Wang et al. (2013). Putting such an idea into practice would not only require study of the roles of other bacteria but also better understanding the role of bacterial viruses (bacteriophage) and amoebae. Evidence that a switch from chlorine disinfection to chloramine resulted in an absence of *Legionella* but an increase in *M. avium* (Baron et al. 2014; Pryor et al. 2004; Williams et al. 2005) illustrates the complexity of the challenge presented by OPPPs and deserves further exploration and research. Studies of the impact of disinfection methods should include the use of ultraviolet irradiation, an agent that not only results in killing but also in mutations. Some studies also indicate that overuse of chlorination can also enhance selection of antibiotic-resistant pathogens (Karumathil et al. 2014; Shrivastava et al. 2004). Because the OPPPs belong to the category of amoeba-resisting microorganisms, a thorough investigation of the role of free-living, phagocytic amoebae in supporting the persistence of OPPPs in premise plumbing is warranted. There is a dynamic, changing relationship between OPPPs and amoebae over time (Buse et al. 2014a). Thus, the interaction between OPPPs and free-living phagocytic amoebae and disinfectant regulation needs careful examination (Buse et al. 2014b; Revetta et al. 2013) to provide a guide for remedial measures. As discussed above concerning remediation and control of OPPPs, it will be important to determine whether microbial ecologic controls could effectively reduce human exposure to OPPPs.

Appendix 1 **Recommended Research Projects Related to Epidemiology and Ecology of OPPPs**

Determine the prevalence, incidence, and trends of disease caused by OPPPs
Determine the dose–response relationship between OPPP numbers and disease
Determine whether the microbiome of a distribution system is a determinant of the premise plumbing microbiome
Determine the role of free-living phagocytic amoebae species on the prevalence, persistence, growth, and survival of OPPPs
Determine whether microbial ecologic controls could reduce exposure to OPPPs
Determine the contributions of bacterial, viral, and eukaryotic microorganisms to the persistence of OPPPs
Determine the impact of disinfection methods, including ultraviolet irradiation, on the emergence of antibiotic-resistant OPPPs.

## References

[r1] Aaron SD, Vandemheen KL, Ramotar K, Giesbrecht-Lewis T, Tullis E, Freitag A (2010). Infection with transmissible strains of *Pseudomonas aeruginosa* and clinical outcomes in adults with cystic fibrosis.. JAMA.

[r2] Al Houqani M, Jamieson F, Chedore P, Mehta M, May K, Marras TK (2011). Isolation prevalence of pulmonary nontuberculous mycobacteria in Ontario in 2007.. Can Respir J.

[r3] Al-Houqani M, Jamieson F, Mehta M, Chedore P, May K, Marras TK (2012). Aging, COPD and other risk factors do not explain the increased prevalence of pulmonary *Mycobacterium avium* complex in Ontario.. Chest.

[r4] Anaissie EJ, Penzak SR, Dignani MC (2002). The hospital water supply as a source of nosocomial infections: a plea for action.. Arch Intern Med.

[r5] Armen RN, Morrow CS (1956). Nontuberculous pulmonary cavitation in anthracosilicosis.. Ann Intern Med.

[r6] Arnow PM, Weil D, Para MF (1985). Prevalence and significance of *Legionella pneumophila* contamination of residential hot-tap water systems.. J Infect Dis.

[r7] Bailey WC, Brown M, Buechner HA, Weill H, Ichinose H, Ziskind M (1974). Silico-mycobacterial disease in sandblasters.. Am Rev Respir Dis.

[r8] Baird SF, Taori SK, Dave J, Willocks LJ, Roddie H, Hanson M (2011). Cluster of non-tuberculous mycobacteraemia associated with water supply in a haemato-oncology unit.. J Hosp Infect.

[r9] Baron J Vikram A Duda S Stout JE Bibby K 2014 Shift in the microbial ecology of a hospital hot water system following the introduction of an on-site monochloramine disinfection system. PLoS One 9 e102679; 10.1371/journal.pone.0102679 25033448 PMC4102543

[r10] Bartrand TA Causey JJ Clancy JL 2014 *Naegleria fowleri*, an emerging drinking water pathogen. J Am Water Works Assoc 106 E418-E432

[r11] Benin AL, Benson RF, Besser RE (2002). Trends in Legionnaires’ disease, 1980–1998: declining mortality and new patterns of diagnosis.. Clin Infect Dis.

[r12] Bentham RH (2000). Routine sampling and the control of *Legionella* spp. in cooling tower water systems.. Curr Microbiol.

[r13] Bertrand X, Bailly P, Blasco G, Balvay P, Boillot A, Talon D (2000). Large outbreak in a surgical intensive care unit of colonization or infection with *Pseudomonas aeruginosa* that overexpressed an active efflux pump.. Clin Infect Dis.

[r14] Blatt SP, Parkinson MD, Pace E, Hoffman P, Dolan D, Lauderdale P (1993). Nosocomial Legionnaires’ disease: aspiration as a primary mode of transmission.. Am J Med.

[r15] Boccia S, Laurenti P, Borella P, Moscato U, Capalbo G, Cambieri A (2006). Prospective three-year surveillance for nosocomial and environmental *Legionella pneumophila*: implications for infection control.. Infect Control Hosp Epidemiol.

[r16] Bodle EE, Cunningham JA, Della-Latta P, Schluger NW, Saiman L (2008). Epidemiology of nontuberculous mycobacteria in patients without HIV infection, New York City.. Emerg Infect Dis.

[r17] Bollin GE, Plouffe JF, Para MF, Hackman B (1985). Aerosols containing *Legionella pneumophila* generated by shower heads and hot-water faucets.. Appl Environ Microbiol.

[r18] Borella P, Montagna MT, Stampi S, Stancanelli G, Romano-Spica V, Triassi M (2005). *Legionella* contamination in hot water of Italian hotels.. Appl Environ Microbiol.

[r19] Bruin JP, Ijzerman EP, den Boer JW, Mouton JW, Diederen BM (2012). Wild-type MIC distribution and epidemiological cut-off values in clinical *Legionella pneumophila* serogroup 1 isolates.. Diagn Microbiol Infect Dis.

[r20] Brooks T, Osicki RA, Springthorpe VS, Satter SA, Filion L, Abrial D (2004). Detection and identification of *Legionella* species from groundwaters.. J Toxicol Environ Health A.

[r21] Brown-Elliott BA, Nash KA, Wallace RJ (2012). Antimicrobial susceptibility testing, drug resistance mechanisms, and therapy of infections with nontuberculous mycobacteria.. Clin Microbiol Rev.

[r22] Buse HY, Lu J, Lu X, Mou X, Ashbolt NJ (2014a). Microbial diversities (16S and 18S rRNA gene pyrosequencing) and environmental pathogens within drinking water biofilms grown on the common premise plumbing materials unplasticized polyvinylchloride and copper.. FEMS Microbiol Ecol.

[r23] Buse HY, Lu J, Struewing IT, Ashbolt NJ (2014b). Preferential colonization and release of *Legionella pneumophila* from mature drinking water biofilms grown on copper versus unplasticized polyvinylchloride coupons.. Int J Hyg Environ Health.

[r24] Butler WR, Sheils CA, Brown-Elliott BA, Charles N, Colin AA, Gant MJ (2007). First isolations of *Segniliparus rugosus* from patients with cystic fibrosis.. J Clin Microbiol.

[r25] Carter G, Wu M, Drummond DC, Bermudez LE (2003). Characterization of biofilm formation by clinical isolates of *Mycobacterium avium.*. J Med Microbiol.

[r26] Castor ML, Wagstrom EA, Danila RN, Smith KE, Naimi TS, Besser JM (2005). An outbreak of Pontiac fever with respiratory distress among workers performing high-pressure cleaning at a sugar-beet processing plant.. J Infect Dis.

[r27] CDC (Centers for Disease Control and Prevention). (2011a). Estimated burden of acute otitis externa–United States, 2003–2007.. MMWR Morb Mortal Wkly Rep.

[r28] CDC (Centers for Disease Control and Prevention). (2011b). Legionellosis—United States, 2000–2009.. MMWR Morb Mortal Wkly Rep.

[r29] CDC (Centers for Disease Control and Prevention). (2013a). Summary of Notifiable Diseases—United States, 2011.. MMWR Morb Mortal Wkly Rep.

[r30] CDC (Centers for Disease Control and Prevention). (2013b). Surveillance for waterborne disease outbreaks associated with drinking water and other nonrecreational water—United States, 2009–2010.. MMWR Morb Mortal Wkly Rep.

[r31] Chen CY, Chen HY, Chou CH, Huang CT, Lai CC, Hsueh PR (2011). Pulmonary infection caused by nontuberculous mycobacteria in a medical center in Taiwan, 2005–2008.. Diagn Microbiol Infect Dis.

[r32] Cholley P, Thouverez M, Floret N, Bertrand X, Talon D (2008). The role of water fittings in intensive care rooms as reservoirs for the colonization of patients with *Pseudomonas aeruginosa.*. Intensive Care Med.

[r33] Cirillo JD, Falkow S, Tompkins LS, Bermudez LE (1997). Interaction of *Mycobacterium avium* with environmental amoebae enhances virulence.. Infect Immun.

[r34] Colbourne JS, Dennis PJ (1989). The ecology and survival of *Legionella pneumophila.*. Water Environ J.

[r35] Collier SA, Stockman LJ, Hicks LA, Garrison LE, Zhou FJ, Beach MJ (2012). Direct healthcare costs of selected diseases primarily or partially transmitted by water.. Epidemiol Infect.

[r36] Collins FM (1989). Mycobacterial disease, immunosuppression, and acquired immunodeficiency syndrome.. Clin Microbiol Rev.

[r37] Cramp GJ, Harte D, Douglas NM, Graham F, Schusboe M, Sykes K (2010). An outbreak of Pontiac fever due to *Legionella longbeachae* serogroup 2 found in potting mix in a horticultural nursery in New Zealand.. Epidemiol Infect.

[r38] Craun GF, Brunkard JM, Yoder JS, Roberts VA, Carpenter J, Wade T (2010). Causes of outbreaks associated with drinking water in the United States from 1971 to 2006.. Clin Microbiol Rev.

[r39] Crivaro V Di Popolo A Caprio A Lambiase A Di Resta M Borriello T 2009 *Pseudomonas aeruginosa* in a neonatal intensive care unit: molecular epidemiology and infection control measures. BMC Infectious Dis 9 70 10.1186/1471-2334-9-70 PMC2692859 19463153

[r40] Cuttelod M, Senn L, Terletskiy V, Nahimana I, Petignat C, Eggimann P (2011). Molecular epidemiology of *Pseudomonas aeruginosa* in intensive care units over a 10-year period (1998–2007).. Clin Microbiol Infect.

[r41] Darelid J, Löfgren S, Malmvall BE (2002). Control of nosocomial Legionnaires’ disease by keeping the circulating hot water temperature above 55°C: experience from a 10-year surveillance programme in a district general hospital.. J Hosp Infect.

[r42] Den Boer JW, Bruin JP, Verhoef LP, Van der Zwaluw K, Jansen R, Yzerman EPF (2008). Genotypic comparison of clinical *Legionella isolates* and patient-related environmental isolates in The Netherlands, 2002–2006.. Clin Microbiol Infect.

[r43] Donlan RM, Costerton JW (2002). Biofilms: survival mechanisms of clinically relevant microorganisms.. Clin Microbiol Rev.

[r44] Donohue MJ, O’Connell K, Vesper SJ, Mistry JH, King D, Kostich M (2014). Widespread molecular detection of *Legionella pneumophila* serogroup 1 in cold water taps across the United States.. Environ Sci Technol.

[r45] Döring G, Hörz M, Ortelt J, Grupp H, Wolz C (1993). Molecular epidemiology of *Pseudomonas aeruginosa* in an intensive care unit.. Epidemiol Infect.

[r46] Ducret A Chabalier M Dukan S 2014 Characterization and resuscitation of ‘non-culturable’ cells of *Legionella pneumophila.* BMC Microbiol 14 3; 10.1186/1471-2180-14-3 24383402 PMC3882098

[r47] du Moulin GC, Sherman IH, Hoaglin DC, Stottmeier KD (1985). *Mycobacterium avium* complex, an emerging pathogen in Massachusetts.. J Clin Microbiol.

[r48] du Moulin GC, Stottmeier KD, Pelletier PA, Tsang AY, Hedley-Whyte J (1988). Concentration of *Mycobacterium avium* by hospital hot water systems.. JAMA.

[r49] Falkinham JO (1996). Epidemiology of infection by nontuberculous mycobacteria.. Clin Microbiol Rev.

[r50] Falkinham JO (2011). Nontuberculous mycobacteria from household plumbing of patients with nontuberculous mycobacteria disease.. Emerg Infect Dis.

[r51] Falkinham JO, Iseman MD, de Haas P, van Soolingen D (2008). *Mycobacterium avium* in a shower linked to pulmonary disease.. J Water Health.

[r52] Falkinham JO, Norton CD, LeChevallier MW (2001). Factors influencing numbers of *Mycobacterium avium, Mycobacterium intracellulare*, and other mycobacteria in drinking water distribution systems.. Appl Environ Microbiol.

[r53] Favero MS, Carson LA, Bond WW, Petersen NJ (1971). *Pseudomonas aeruginosa*: growth in distilled water from hospitals.. Science.

[r54] Feazel LM, Baumgartner LK, Peterson KL, Frank DK, Harris JK, Pace NR (2009). Opportunistic pathogens enriched in showerhead biofilms.. Proc Nat Acad Sci USA.

[r55] Fowler SJ, French J, Screaton NJ, Foweracker J, Condiffe A, Haworth CS (2006). Nontuberculous mycobacteria in bronchiectasis: prevalence and patient characteristics.. Eur Respir J.

[r56] Fraser DW, Tsai TR, Orenstein W, Parkin WE, Beecham HJ, Sharrar RG (1977). Legionnaires’ disease—description of an epidemic of pneumonia.. N Eng J Med.

[r57] Fujita J, Kishimoto T, Ohtsuki Y, Shigito E, Ohnishi T, Shiode M (2004). Clinical features of eleven cases of *Mycobacterium avium–intracellulare* complex pulmonary disease associated with pneumoconiosis.. Respir Med.

[r58] Fujitani S, Sun HY, Yu VL, Weingarten JA (2011). Pneumonia due to *Pseudomonas aeruginosa*. part 1: epidemiology, clinical diagnosis, and source.. Chest.

[r59] Garcia MT, Pelaz C (2008). Effectiveness of disinfectants used in cooling towers against *Legionella pneumophila.*. Chemotherapy.

[r60] Gaynes R, Edwards JR, National Nosocomial Infections Surveillance System (2005). Overview of nosocomial infections caused by gram-negative bacilli.. Clin Infect Dis.

[r61] Geddes D (2008). Segregation is not good for patients with cystic fibrosis.. J R Soc Med.

[r62] Griffith DE, Aksamit T, Brown-Elliott BA, Catanzaro A, Daley C, Gordin F (2007). An official ATS/IDSA statement: diagnosis, treatment, and prevention of nontuberculous mycobacterial diseases.. Am J Respir Crit Care Med.

[r63] Grobe S, Wingender J, Flemming HC (2001). Capability of mucoid *Pseudomonas aeruginosa* to survive in chlorinated water.. Int J Hyg Environ Health.

[r64] Gubler JG, Salfinger M, von Graevenitz A (1992). Pseudoepidemic of nontuberculous mycobacteria due to a contaminated bronchoscope cleaning machine. Report of an outbreak and review of the literature.. Chest.

[r65] Hardalo C, Edberg SC (1997). *Pseudomonas aeruginosa:* assessment of risk from drinking water.. Crit Rev Microbiol.

[r66] Hayes D, West SE, Rock MJ, Li ZL, Splaingard ML, Farrell PM (2010). *Pseudomonas aeruginosa* in children with cystic fibrosis diagnosed through newborn screening: assessment of clinic exposures and microbial genotypes.. Pediatr Pulmonol.

[r67] Hilborn ED, Covert TC, Yakrus MA, Harris SI, Donnelly SF, Rice EW (2006). Persistence of nontuberculous mycobacteria in a drinking water system after addition of filtration treatment.. Appl Environ Microbiol.

[r68] Hilborn ED, Yakrus MA, Covert TC, Harris SI, Donnely SF, Schmitt MT (2008). Molecular comparison of *Mycobacterium avium* isolates from clinical and environmental sources.. Appl Environ Microbiol.

[r69] Hlavsa MC, Roberts VA, Kahler AM, Hilborn ED, Wade TJ, Backer LC (2014). Recreational water-associated disease outbreaks—United States, 2009–2010.. MMWR Morb Mortal Wkly Rep.

[r70] Hoebe CJP, Cluitmans JJM, Wagenvoort JHT (1998). Two fatal cases of nosocomial *Legionella pneumophila* pneumonia associated with a contaminated cold water supply.. Eur J Clin Microbiol Infect Dis.

[r71] Høiby N, Ciofu O, Bjarnsholt T (2010). *Pseudomonas aeruginosa* biofilms in cystic fibrosis.. Future Microbiol.

[r72] Holinger EP, Ross KA, Robertson CE, Stevens MJ, Harris JK, Pace NR (2014). Molecular analysis of point-of-use municipal drinking water microbiology.. Water Res.

[r73] Holland SM (2007). Interferon gamma, IL-12, IL-12R and STAT-1 immunodeficiency diseases: disorders of the interface of innate and adaptive immunity.. Immunol Res.

[r74] Hollyoak V, Boyd P, Freeman R (1995). Whirlpool baths in nursing homes: use, maintenance, and contamination with *Pseudomonas aeruginosa.*. Commun Dis Rep CDR Rev.

[r75] Horsburgh CR, Selik RM (1989). The epidemiology of disseminated nontuberculous mycobacterial infection in the acquired immunodeficiency syndrome (AIDS).. Am Rev Respir Dis.

[r76] Hosein IK, Hill DW, Tan TY, Butchart EG, Wilson K, Finlay G (2005). Point-of care controls for nosocomial legionellosis combined with chlorine dioxide potable water decontamination: a two year survey at a Welsh teaching hospital.. J Hosp Infect.

[r77] Hsu SC, Martin R, Wentworth BB (1984). Isolation of *Legionella* species from drinking water.. Appl Environ Microbiol.

[r78] Hung LL, Copperthite DC, Yang CS, Lewis FA, Zampiello FA (1993). Environmental *Legionella* assessment in office buildings of continental United States.. Indoor Air.

[r79] Iseman MD, Buschman DL, Ackerson LM (1991). Pectus excavatum and scoliosis. Thoracic anomalies associated with pulmonary disease caused by *Mycobacterium avium* complex.. Am Rev Respir Dis.

[r80] Joseph CA, Watson JM, Harrison TG, Bartlett CLR (1994). Nosocomial Legionnaires’ disease in England and Wales, 1980-92.. Epidemiol Infect.

[r81] Kahana LM, Kay JM, Yakrus MA, Waserman S (1997). *Mycobacterium avium* complex infection in an immunocompetent young adult related to hot tub exposure.. Chest.

[r82] Karumathil DP, Yin HB, Kollanoor-Johny A, Venkitanarayanan K (2014). Effect of chlorine exposure on the survival and antibiotic gene expression of multidrug resistant *Acinetobacter baumannii* in water.. Int J Environ Res Public Health.

[r83] Kilvington S, Price J (1990). Survival of *Legionella pneumophila* within cysts of *Acanthamoeba polyphaga* following chlorine exposure.. J Appl Bacteriol.

[r84] Kim BR, Anderson JE, Mueller SA, Gaines WA, Kendall AM (2002). Literature review—efficacy of various disinfectants against *Legionella* in water systems.. Water Res.

[r85] Kim RD, Greenberg DE, Ehrmantraut ME, Guide SV, Ding L, Shea Y (2008). Pulmonary nontuberculous mycobacterial disease: prospective study of a distinct preexisting syndrome.. Am J Respir Crit Care Med.

[r86] Klockgether J Cramer N Wiehlmann L Davenport CF Tümmler B 2011 *Pseudomonas aeruginosa* genomic structure and diversity. Front Microbiol 2 150; 10.3389/fmicb.2011.00150 21808635 PMC3139241

[r87] Kool JL, Bergmire-Sweat D, Butler JC, Brown EW, Peabody DJ, Massi DS (1999). Hospital characteristics associated with colonization of water systems by *Legionella* and risk of nosocomial legionnaires’ disease: a cohort study of 15 hospitals.. Infect Control Hosp Epidemiol.

[r88] Kozak-Muiznieks NA, Lucas CE, Brown E, Pondo T, Taylor TH, Frace M (2014). Prevalence of sequence types among clinical and environmental isolates of *Legionella pneumophila* serogroup 1 in the United States from 1982 to 2012.. J Clin Microbiol.

[r89] Kozicki ZA, Cwiek MA, Lopes JE, Rodabaugh G, Tymes N, Baiyasi-Kozicki JS (2012). Waterborne pathogens: a public health risk in US hospitals.. J Am Water Works Assoc.

[r90] Kusnetsov J, Torvinen E, Perola O, Nousiainen T, Katila ML (2003). Colonization of hospital water systems by legionellae, mycobacteria and other heterotrophic bacteria potentially hazardous to risk group patients.. APMIS.

[r91] Lanini S D’Arezzo S Puro V Martini L Imperi F Piselli P 2011 Molecular epidemiology of a *Pseudomonas aeruginosa* hospital outbreak driven by a contaminated disinfectant-soap dispenser. PloS One 6 e17064; 10.1371/journal.pone.0017064 21359222 PMC3040201

[r92] Lin YE, Vidic RD, Stout JE, Yu VL (1998). *Legionella* in water distribution systems.. J Am Water Works Assoc.

[r93] Marras TK, Daley CL (2002). Epidemiology of human pulmonary infection with nontuberculous mycobacteria.. Clin Chest Med.

[r94] Marras TK, Mendelson D, Marchand-Austin A, May K, Jamieson FB (2013). Pulmonary nontuberculous mycobacterial disease, Ontario, Canada, 1998–2010.. Emerg Infect Dis.

[r95] Masák J, Cejková A, Schreiberová O, Rezanka T (2014). *Pseudomonas* biofilms: possibilities of their control.. FEMS Microbiol Ecol.

[r96] Maugein J, Dailloux M, Carbonnelle B, Vincent V, Grosset J, French Mycobacteria Study Group (2005). Sentinel-site surveillance of *Mycobacterium avium* complex pulmonary disease.. Eur Respir J.

[r97] Mena KD, Gerba CP (2009). Risk assessment of *Pseudomonas aeruginosa* in water.. Rev Environ Contam Toxicol.

[r98] Mullis SN, Falkinham JO (2013). Adherence and biofilm formation of *Mycobacterium avium, Mycobacterium intracellulare* and *Mycobacterium abscessus* to household plumbing materials.. J Appl Microbiol.

[r99] Murga R, Forster TS, Brown E, Pruckler JM, Fields BS, Donlan RM (2001). Role of biofilms in the survival of *Legionella pneumophila* in a model potable-water system.. Microbiology.

[r100] Neil K, Berkelman R (2008). Increasing incidence of legionellosis in the United States, 1990–2005: changing epidemiological trends.. Clin Infect Dis.

[r101] Niederman MS, Mandell LA, Anzueto A, Bass JB, Broughton WA, Campbell GD (2001). Guidelines for the management of adults with community-acquired pneumonia. Diagnosis, assessment of severity, antimicrobial therapy, and prevention.. Am J Respir Crit Care Med.

[r102] Nguyen TMN, Ilef D, Jarraud S, Rouil L, Campese C, Che D (2006). A community-wide outbreak of legionnaires’ disease linked to industrial cooling towers—how far can contaminated aerosols spread?. J Infect Dis.

[r103] Norton CD, LeChevallier MW, Falkinham JO (2004). Survival of *Mycobacterium avium* in a model distribution system.. Water Res.

[r104] Olivier KN, Weber DJ, Wallace RJ, Faiz AR, Lee JH, Zhang Y (2003). Nontuberculous mycobacteria. I. Multicenter prevalence study in cystic fibrosis.. Am J Respir Crit Care Med.

[r105] O’Loughlin RE Kightlinger L Werpy MC Brown E Stevens V Hepper C 2007 Restaurant outbreak of Legionnaires’ disease associated with a decorative fountain: an environmental and case-control study. BMC Infect Dis 7 93; 10.1186/1471-2334-7-93 17688692 PMC1976126

[r106] Perkins SD, Mayfield J, Fraser V, Angenent LT (2009). Potentially pathogenic bacteria in shower water and air of a stem cell transplant unit.. Appl Environ Microbiol.

[r107] Phares CR, Russell E, Thigpen MC, Service W, Crist MB, Salyers M (2007). Legionnaires’ disease among residents of a long-term care facility: the sentinel event in a community outbreak.. Am J Infect Control.

[r108] Philley JV, Griffith DE (2013). Management of nontuberculous mycobacterial (NTM) lung disease.. Semin Respir Crit Care Med.

[r109] Piersimoni C, Scarparo C (2009). Extrapulmonary infections associated with nontuberculous mycobacteria in immunocompetent persons.. Emerg Infect Dis.

[r110] Polverino E, Dambrava P, Cillóniz C, Balasso V, Marcos MA, Esquinas C (2010). Nursing home-acquired pneumonia: a 10 year single-centre experience.. Thorax.

[r111] Prevots DR, Shaw PA, Strickland D, Jackson LA, Raevel MA, Blosky MA (2010). Nontuberculous mycobacterial lung disease prevalence at four integrated health care delivery systems.. Am J Resp Crit Care Med.

[r112] Prince DS, Peterson DD, Steiner RM, Gottlieb JE, Scott R, Israel HL (1989). Infection with *Mycobacterium avium* complex in patients without predisposing conditions.. N Engl J Med.

[r113] Pruden A, Edwards MA, Falkinham JO III. (2013). State of the Science and Research Needs for Opportunistic Pathogens in Premise Plumbing. Water Research Foundation Project 4379 Final Report. Denver, CO:Water Research Foundation.. http://www.waterrf.org/Pages/Projects.aspx?PID=4379.

[r114] Pryor M, Springthorpe S, Riffard S, Brooks T, Huo Y, Davis G (2004). Investigation of opportunistic pathogens in municipal drinking water under different supply and treatment regimes.. Water Sci Technol.

[r115] Ragull S, Garcia-Nuñez M, Pedro-Botet ML, Sopena N, Esteve M, Montenegro R (2007). *Legionella pneumophila* in cooling towers: fluctuations in counts, determination of genetic variability by pulsed-field gel electrophoresis (PFGE) and persistence of PFGE patterns.. Appl Environ Microbiol.

[r116] Rajan S, Saiman L (2002). Pulmonary infections in patients with cystic fibrosis.. Sem Respir Infect.

[r117] Reuter S, Sigge A, Wiedeck H, Trautmann M (2002). Analysis of transmission pathways of *Pseudomonas aeruginosa* between patients and tap water outlets.. Crit Care Med.

[r118] Revetta RP, Gomez-Alvarez V, Gerke TL, Curioso C, Santo Domingo JW, Ashbolt NJ (2013). Establishment and early succession of bacterial communities in monochloramine-treated drinking water biofilms.. FEMS Microbiol Ecol.

[r119] Rhoads WJ, Pruden A, Edwards MA (2014). Anticipating challenges with in-building disinfection for control of opportunistic pathogens.. Water Environ Res.

[r120] Rodgers MR, Blackstone BJ, Reyes AL, Covert TC (1999). Colonisation of point of use water filters by silver resistant non-tuberculous mycobacteria [Letter].. J Clin Pathol.

[r121] Rogues AM, Boulestreau H, Lashéras A, Boyer A, Gruson D, Merle C (2007). Contribution of tap water to patient colonisation with *Pseudomonas aeruginosa* in a medical intensive care unit.. J Hosp Infect.

[r122] Russell AD (1999). Bacterial resistance to disinfectants: present knowledge and future problems.. J Hosp Infect.

[r123] Ryoo SW, Shin S, Shim MS, Park YS, Lew WJ, Park SN (2008). Spread of nontuberculous mycobacteria from 1993 to 2006 in Koreans.. J Clin Lab Anal.

[r124] Sabria M, Alvarez J, Dominguez A, Pedrol A, Sauca G, Salleras L (2006). A community outbreak of Legionnaires’ disease: evidence of a cooling tower as the source.. Clin Microbiol Infect.

[r125] Sabrià M, García-Nuñez M, Pedro-Botet ML, Sopena N, Gimeno JM, Reynaga E (2001). Presence and chromosomal subtyping of *Legionella* species in potable water systems in 20 hospitals of Catalonia, Spain.. Infect Control Hosp Epidemiol.

[r126] Schulze-Röbbecke R, Janning B, Fischeder R (1992). Occurrence of mycobacteria in biofilm samples.. Tuber Lung Dis.

[r127] Schuster FL, Visvesvara GS (2004). Free-living amoebae as opportunistic and non-opportunistic pathogens of humans and animals.. Int J Parasitol.

[r128] Seyfried PL, Fraser DJ (1980). Persistence of *Pseudomonas aeruginosa* in chlorinated swimming pools.. Can J Microbiol.

[r129] Shrivastava R, Upreti RK, Jain SR, Prasad KN, Seth PK, Chaturvedi UC (2004). Suboptimal chlorine treatment of drinking water leads to selection of multidrug-resistant *Pseudomonas aeruginosa.*. Ecotoxicol Environ Saf.

[r130] Simões LC, Simões M, Vieira MJ (2010). Influence of the diversity of bacterial isolates from drinking water on resistance of biofilms to disinfection.. Appl Environ Microbiol.

[r131] Smirnov A, Nassonova E, Berney C, Fahrni J, Bolivar I, Pawlowski J (2005). Molecular phylogeny and classification of the lobose amoebae.. Protist.

[r132] Sonnenberg P, Murray J, Glynn JR, Thomas RG, Godfrey-Faussett P, Shearer S (2000). Risk factors for pulmonary disease due to culture-positive *M. tuberculosis* or nontuberculous mycobacteria in South African gold miners.. Eur Respir J.

[r133] Squier C, Yu VL, Stout JE (2000). Waterborne nosocomial infections.. Curr Infect Dis Rep.

[r134] States SJ, Conley LF, Kuchta JM, Oleck BM, Lipovich MJ, Wolford RS (1987). Survival and multiplication of *Legionella pneumophila* in municipal drinking water systems.. Appl Environ Microbiol.

[r135] Steed KA, Falkinham JO (2006). Effect of growth in biofilms on chlorine susceptibility of *Mycobacterium avium* and *Mycobacterium intracellulare.*. Appl Environ Microbiol.

[r136] Stout JE, Muder RR (2004). *Legionella* in residential water systems.. ASHRAE J.

[r137] Stout JE, Yu VL (2003). Hospital-acquired Legionnaires’ disease: new developments.. Curr Opin Infect Dis.

[r138] Stout JE, Yu VL (1997). Legionellosis.. N Engl J Med.

[r139] Stout JE, Yu VL, Best MG (1985). Ecology of *Legionella pneumophila* within water distribution systems.. Appl Env Microbiol.

[r140] Suman E, Singh S, Kotian S (2008). *Pseudomonas aeruginosa* biofilms in hospital water systems and the effect of sub-inhibitory concentrations of chlorine.. J Hosp Infect.

[r141] Tablan OC Anderson LJ Besser R Bridges C Haijeh R, CDC (Centers for Disease Prevention and Control), et al. 2004 Guidelines for preventing health-care–associated pneumonia, 2003: recommendations of the CDC and the Healthcare Infection Control Practices Advisory Committee. MMWR Recomm Rep 53 RR-3 1 36 15048056

[r142] Taylor RH, Falkinham JO, Norton CD, LeChevallier MW (2000). Chlorine, chloramine, chlorine dioxide, and ozone susceptibility of *Mycobacterium avium.*. Appl Environ Microbiol.

[r143] Temmerman R, Vervaeren H, Noseda B, Boon N, Verstraete W (2006). Necrophilic growth of *Legionella pneumophila.*. Appl Environ Microbiol.

[r144] Thomas JM, Ashbolt NJ (2011). Do free-living amoebae in treated drinking water systems present an emerging health risk?. Environ Sci Technol.

[r145] Thomson R, Carter R, Gilpin C, Coulter C, Hargreaves M (2008). Comparison of methods for processing drinking water samples for the isolation of *Mycobacterium avium* and *Mycobacterium intracellulare.*. Appl Environ Microbiol.

[r146] Tobin-D’Angelo MJ, Blass MA, del Rio C, Halvosa JS, Blumberg HM, Horsburgh CR (2004). Hospital water as a source of *Mycobacterium avium* complex isolates in respiratory specimens.. J Infect Dis.

[r147] Tortoli E (2003). Impact of genotype studies on mycobacterial taxonomy: the new mycobacteria of the 1990s.. Clin Microbiol Rev.

[r148] Trautmann M, Bauer C, Schumann C, Hahn P, Höher P, Haller M (2009). Common RAPD pattern of *Pseudomonas aeruginosa* from patients and tap water in a medical intensive care unit.. Int J Hyg Environ Health.

[r149] Trautmann M, Lepper PM, Haller M (2005). Ecology of *Pseudomonas aeruginosa* in the intensive care unit and the evolving role of water outlets as a reservoir of the organism.. Am J Infect Control.

[r150] Trautmann M, Michalsky T, Wiedeck H, Radosavljevic V, Ruhnke M (2001). Tap water colonization with *Pseudomonas aeruginosa* in a surgical intensive care unit (ICU) and relation to *Pseudomonas* infections of ICU patients.. Infect Control Hosp Epidemiol.

[r151] Tsintzou A, Vantarakis A, Pagonopoulou O, Athanassiadou A, Papapetropoulou M (2000). Environmental mycobacteria in drinking water before and after replacement of the water distribution network.. Water Air Soil Pollut.

[r152] Tsukamura M, Kita N, Shimoide H, Arakawa H, Kuze A (1988). Studies on the epidemiology of nontuberculous mycobacteriosis in Japan.. Am Rev Respir Dis.

[r153] United Nations Population Division. (2002). World Population Ageing: 1950–2050. New York:United Nations.. http://www.globalaging.org/ruralaging/world/ageingo.htm.

[r154] U.S. Environmental Protection Agency. (2010). Disinfection. 40 CFR Ch. I (7–1–10 Edition), § 141.72. http://www.gpo.gov/fdsys/pkg/CFR-2010-title40-vol22/pdf/CFR-2010-title40-vol22-sec141-72.pdf.

[r155] Vaz-Moreira I, Nunes OC, Manaia CM (2012). Diversity and antibiotic resistance in *Pseudomonas* spp. from drinking water.. Sci Tot Environ.

[r156] von Reyn CF, Maslow JN, Barber TW, Falkinham JO, Arbeit RD (1994). Persistent colonisation of potable water as a source of *Mycobacterium avium* infection in AIDS.. Lancet.

[r157] Wallace RJ, Brown BA, Griffith DE (1998). Nosocomial outbreaks/pseudo-outbreaks caused by nontuberculous mycobacteria.. Annu Rev Microbiol.

[r158] Wang H, Edwards M, Falkinham JO, Pruden A (2013). Probiotic approach to pathogen control in premise plumbing systems? A review.. Environ Sci Technol.

[r159] Wang HX, Yue J, Han M, Yang JH, Gao RL, Jing LJ (2010). Nontuberculous mycobacteria: susceptibility pattern and prevalence rate in Shanghai from 2005 to 2008.. Chin Med J (Engl).

[r160] Williams MM, Chen TH, Keane T, Toney N, Toney S, Armbruster CR (2011). Point-of-use membrane filtration and hyperchlorination to prevent patient exposure to rapidly growing mycobacteria in the potable water supply of a skilled nursing facility.. Infect Contr Hosp Epidemiol.

[r161] Williams MM, Santo Domingo JW, Meckes MC (2005). Population diversity in model water biofilms receiving chlorine or chloramine residual.. Biofouling.

[r162] Williams MM, Yakrus MA, Arduino MJ, Cooksey RC, Crane CB, Banerjee SN (2009). Structural analysis of biofilm formation by rapidly and slowly growing nontuberculous mycobacteria.. Appl Environ Microbiol.

[r163] Winthrop KL, Abrams M, Yakrus M, Schwartz I, Ely J, Gillies D (2002). An outbreak of mycobacterial furunculosis associated with footbaths at a nail salon.. N Engl J Med.

[r164] Winthrop KL, McNelley E, Kendall B, Marshall-Olson A, Morris C, Cassidy M (2010). Pulmonary nontuberculous mycobacterial disease prevalence and clinical features: an emerging public health disease.. Am J Respir Crit Care Med.

[r165] Witty LA, Tapson VF, Piantadosi CA (1994). Isolation of mycobacteria in patients with pulmonary alveolar proteinosis.. Medicine (Baltimore).

[r166] Wolinsky E (1995). Mycobacterial lymphadenitis in children: a prospective study of 105 nontuberculous cases with long-term follow-up.. Clin Infect Dis.

[r167] Yamazaki Y, Danelishvili L, Wu M, Hidaka E, Katsuyama T, Stang B (2006). The ability to form biofilm influences *Mycobacterium avium* invasion and translocation of bronchial epithelial cells.. Cell Microbiol.

[r168] Yoder JS, Hlavsa MC, Craun GF, Hill V, Roberts V, Yu PA (2008). Surveillance for waterborne disease and outbreaks associated with recreational water use and other aquatic facility-associated health events—United States, 2005–2006.. MMWR Surveill Summ.

[r169] Yu VL (1993). Could aspiration be the major mode of transmission for *Legionella*?. Am J Med.

[r170] Zhang Z, McCann C, Stout JE, Piescznski S, Hawks R, Vidic R (2007). Safety and efficacy of chlorine dioxide for *Legionella* control in a hospital water system.. Infect Control Hosp Epidemiol.

